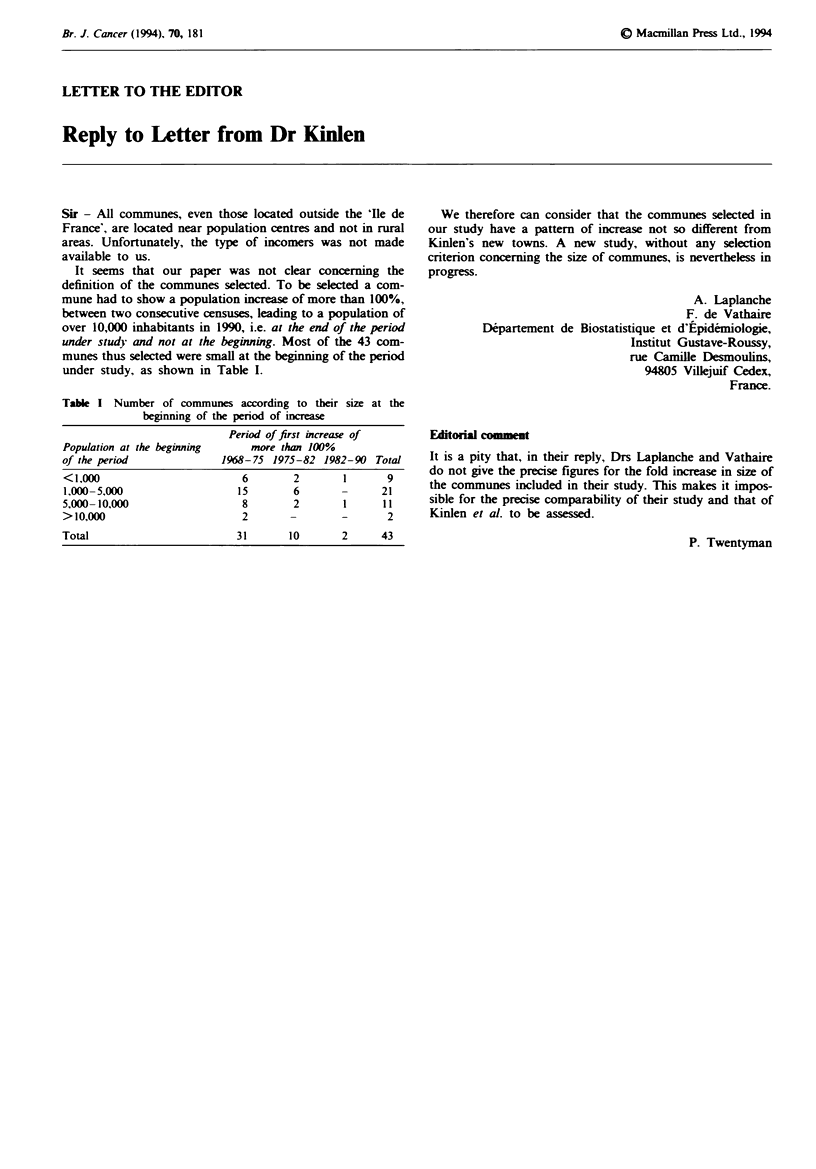# Reply to Letter from Dr Kinlen

**Published:** 1994-07

**Authors:** A. Laplanche, F. de Vathaire


*Br. J. Cancer* (1994), **70**, 181 © Macmillan Press Ltd., 1994

### LETTER TO THE EDITOR

### Reply to Letter from Dr Kinlen

**Sir** – All communes, even those located outside the 'Ile de France', are located near population centres and not in rural areas. Unfortunately, the type of incomers was not made available to us.

It seems that our paper was not clear concerning the definition of the communes selected. To be selected a commune had to show a population increase of more than 100%, between two consecutive censuses, leading to a population of over 10,000 inhabitants in 1990, i.e. *at the end of the period under study and not at the beginning*. Most of the 43 communes thus selected were small at the beginning of the period under study, as shown in Table I.

**Table I** Number of communes according to their size at the beginning of the period of increase

| *Population at the beginning of the period* | *Period of first increase of more than 100%* 1968–75 | 1975–82 | 1982–90 | *Total* |
|---|---|---|---|---|
| <1,000 | 6 | 2 | 1 | 9 |
| 1,000–5,000 | 15 | 6 | – | 21 |
| 5,000–10,000 | 8 | 2 | 1 | 11 |
| >10,000 | 2 | – | – | 2 |
| Total | 31 | 10 | 2 | 43 |

We therefore can consider that the communes selected in our study have a pattern of increase not so different from Kinlen's new towns. A new study, without any selection criterion concerning the size of communes, is nevertheless in progress.

A. Laplanche
F. de Vathaire
Département de Biostatistique et d'Épidémiologie,
Institut Gustave-Roussy,
rue Camille Desmoulins,
94805 Villejuif Cedex,
France.

**Editorial comment**

It is a pity that, in their reply, Drs Laplanche and Vathaire do not give the precise figures for the fold increase in size of the communes included in their study. This makes it impossible for the precise comparability of their study and that of Kinlen *et al.* to be assessed.

P. Twentyman